# Smart Non-Woven Fiber Mats with Light-Induced Sensing Capability

**DOI:** 10.3390/nano10010077

**Published:** 2019-12-31

**Authors:** Igor Krupa, Patrik Sobolčiak, Miroslav Mrlik

**Affiliations:** 1 Center for Advanced Materials, Qatar University, Doha P.O. Box 2713, Qatar; patrik@qu.edu.qa; 2 Centre of Polymer Systems, University Institute, Tomas Bata University in Zlin, Trida T. Bati 5678, 76001 Zlin, Czech Republic

**Keywords:** light-induced actuation, PBMA, PVDF-*co*-HFP, graphene oxide, elastomers, sensing

## Abstract

This article is focused on the facile procedure for 2D graphene oxide (GO) fabrication, utilizing reversible de-activation polymerization approach and therefore enhanced compatibility with surrounding polymer matrix. Such tunable improvement led to a controllable sensing response after irradiation with light. The neat GO as well as surface initiated atom transfer radical polymerization (SI-ATRP) grafted particles were investigated by atomic force microscopy, Fourier transform infrared spectroscopy and thermogravimetric analysis. To confirm the successful surface reduction, X-ray photoelectron spectroscopy and Raman spectroscopy was utilized. The composites in form of non-woven fiber mats containing ungrafted GO and controllably grafted GO with compact layer of polymer dispersed in poly(vinylidene-*co*-hexafluoropropylene) were prepared by electrospinning technique and characterized by scanning electron microscopy. Mechanical performance was characterized using dynamic mechanical analysis. Thermal conductivity was employed to confirm that the conducting filler was well-dispersed in the polymer matrix. The presented controllable coating with polymer layer and its impact on the overall performance, especially photo-actuation and subsequent contraction of the material aiming on the sensing applications, was discussed.

## 1. Introduction

Smart systems belong to the group of materials capable of changing the basic properties, when they are exposed to external stimuli such as electric [1,2,3], magnetic [4,5], thermal [6,7], pH [8,9], or light [10,11]. In case of light stimulation, such smart systems can exhibit the shape [12] or resistivity [13] change or generate electric output [14].

Generally, photo-actuating systems can be classified as composites containing two phases. The filler absorbs the light of certain wavelength while the matrix exhibits appropriate elasticity. The majority of the fillers are based on carbon (carbon nanotubes [15,16] (CNTs) or graphene particles [17] and more specifically based on graphene oxide [18,19] (GO)). There are also some other substances with photo-active capability such as azobenzene-based molecules [20,21] that are utilized as well. In case of matrices, liquid crystals are the most applied materials [16,22,23,24,25,26]. In the case of chemically cross-linked systems, poly(dimethyl siloxanes) [27,28] are used. The thermoplastic elastomers i.e., TPU [12], the most frequently used being block copolymer elastomers styrene-*co*-isoprene-*co*-styrene [29] (SIS) and poly(methyl methacrylate)-*co*-poly(butyl acrylate)-*co*-poly(methyl methacrylate) (PMMA-PBA-PMMA) [30] triblock copolymers, have been utilized and showed excellent performance. The photo-actuating performance can be effectively applied in many applications, i.e., smart displays for visually impaired people [31], smart curtains [32], or caterpillar movement [33].

The utilization of the PVDF-based materials filled by various particle systems like graphene oxide [34], cellulose [35], or spider silk [36] lead to enhancement its piezo-activity. This approach also finds the utilization for sensing applications with the help of another PVDF-based systems like PVDF-*co*-HFP [37]. Also, the application of the electrospinning process for further fabrication is a useful tool for improving the electro-activity of this system and shows proper mechanical performance of the final fiber mats [38] or specifically printed structures using melt-electro writing [39].

This work provides the system with good mechanical properties, where dispersibility is a crucial factor. Processability in a large scale, together with photo-actuation performance, as well as significant change of the system resistivity upon deformation is also a very important factor, similarly as was shown elsewhere [40] where the conductivity of this composite system plays an important role [41].

Thus, the presented article shows simple fabrication of the smart composites with controllably coated and reduced GO with a polymer shell [42], and poly(vinylidene-*co*-hexafluoropropylene) PVDF-*co*-HFP non-woven mats. The PVDF-*co*-HFP was used because of its excellent mechanical properties after electrospinning in the form of fibers with enormous elasticity achieving proper actuation performance upon photo-stimulation [38].

## 2. Materials and Methods

Graphite (powder, <20 μm), sodium nitrate (NaNO_3_, ≥99%), sulphuric acid (H_2_SO_4_, reagent grade, 95–98%), hydrogen peroxide (29.0–32.0 wt %), and potassium permanganate (KMnO4, 97%). The α-bromoisobutyryl bromide (BiBB, 98%), triethyleneamine (TEA, ≥99%). Ethyl α-bromoisobutyrate (EBiB, 98%), anisole (99%), butyl methacrylate (BMA, 99%), N,N,N′,N″,N″-pentamethyldiethylenetriamine (PMDETA, ≥99%), diethyl ether (anhydrous, ≥99%), and copper bromide (CuBr, ≥99%). All chemicals were purchased from Sigma Aldrich (St. Louis, MO, USA). BMA was purified by neutral alumina column to remove MEHQ inhibitor. Tetrahydrofurane (anhydrous, THF, p.a.), dimethylformamide (DMF, p.a.), acetone (p.a.), diethyl ether (p.a.), and hydrochloric acid (HCl, 35%, p.a.) were all from Penta Labs (Brno, Czech Republic), poly(vinylidene-*co*-hexafrluoropropylene) (PVDF-*co*-HFP) Mn = 130,000 g·mol^−1^ was purchased from Sigma Aldrich (St. Louis, MO, USA) and used as received.

### 2.1. Graphene Oxide Fabrication and Immobilization of Initiator on Surface

The modified Hummer’s method was used for fabrication of the graphene oxide (GO) sheets precisely described by Osicka et al. [43]. Dried GO particles (2 g) were evacuated in a three-neck round bottom flask and hydroxyl groups were linked with BiBB initiator (7 mL) in the presence of THF (60 mL) and TEA (12 mL) under inert argon atmosphere and upon esterification conditions at 5 °C. Final purification was performed by washing with THF (50 mL) and acetone (50 mL) three times using filtration apparatus and finally with diethyl ether 50 mL and placed under vacuum at 30 °C for 6 h.

### 2.2. Grafting of GO with Poly(N-Butyl Methacrylate) (PBMA) Chains

Graphene oxide (0.75 g) was placed to Schlenk flask (SF) and three-times evacuated and filled with inert atmosphere. Then, EBiB (1.0995 mmol), BMA (109.95 mmol), anisole (50 vol %), and PMDETA (4.398 mmol) were step by step added to the SF. To avoid the present of oxygen in the system the freeze–pump–thaw cycles were applied four times and followed by addition of CuBr (1.0995 mmol). Reaction was carried out for 2 h at 60 °C. Reaction was stopped by SF open and reaction mixture was filtered by DMF (150 mL), acenote (150 mL) two times, and by diethyl ether (200 mL), and dried in a vacuum at 20 mbar and 40 °C overnight.

### 2.3. Elastomeric Composite Preparation via Electrospinning Process

Electrospinning process was performed on the solutions of neat PVDF-*co*-HFP and DMF in rations of 2 to 8 and in case of systems based on GO and GO-PBMA by mixing of 0.1 wt % of particles inside the solution. The solutions were sonicated at an amplitude of 0.6 for 1 h followed by magnetic stirring for 4 h at ambient temperature. Then, the solutions were pressed through the syringe and an 18-gauge needle 5 cm apart from the collector at 12 kV. Final electrospun mats were dried in a vacuum at 30 °C overnight.

### 2.4. General Characterization of the Synthesized Powders and Prepared Composites

The molar mass and polydispersity (PDI) of PBMA chains were investigated using gel permeation chromatography (GPC) on the GPC instrument (PL-GPC220, Agilent, Tokyo, Japan) at 30 °C and PS as standard. ^1^H nuclear magnetic resonance (NMR) spectra were recorded at 25 °C, (400 MHz VNMRS Varian, Tokyo, Japan). Fourier transform infrared (FTIR) spectra (64 scans, resolution of 4 cm^−1^) were recorded on a Nicolet 6700 (Nicolet, Watertown, MA, USA) in the range of 600–3600 cm^−1^. The Raman spectra (3 scans, resolution of 2 cm^−1^) were collected on a Nicolet DXR (Nicolet, Green Bay, WI, USA) using an excitation wavelength of 532 nm. Atomic force microscopy (AFM) of GO-sheets were performed by AFM, Dimension Icon Bruker, Karlsruhe, Germany. Size of the individual fibers in the mats were elucidated using scanning electron microscope (SEM) (Tescan-VEGA II, Brno, Czech Republic). The XPS analysis chamber was evacuated approximately 6 × 10^−8^ Pa. The samples were excited by X-ray over a 400 µm^2^ spot area with monochromatic Al *Kα*1,2 radiation at 1486.6 eV. All the spectra were referenced to the main C1s peak of the carbon atoms, which was assigned a value of 284.8 eV. The viscoelastic properties samples were studied in tensile mode. Samples were investigated at the linear viscoelastic region (1 Hz, 0.1% strain) at temperature sweep from −150 °C to 150 °C (DMA 1, Mettler Toledo, Zurich, Switzerland). Samples for electrical conductivity in the form of powder were pressed on the hydraulic press (H-62, Trystom, Olomouc, Czech Republic). Then, current responses were evaluated using electrometer (Keithley 6517B, Cleveland, TX, USA) and 10 measurements were used to provide the final value. Contact angle measurement (CA) was evaluated from the static sessile drop method carried out on a surface energy evaluation system equipped with a CCD camera (Advex Instruments, Brno, Czech Republic). The drops of 5 μL were placed on the GO substrates and the final value of contact angle was averaged from 5 individual measurements.

### 2.5. Photo-Actuation and Sensing Performance

In order to investigate the photo-responsive properties of neat polymer matrix and prepared nanocomposites in the form of electrospun mats, the thermo-mechanical analyzer (TMA) (Mettler Toledo, Columbus, OH, USA) has been used [16]. The photo-actuation performance ability of the material shows the reversible contraction and elongation upon irradiation with light source having 627 nm wavelength. To properly collect the data from sensing capability, the samples were decorated with copper electrodes using vacuum evaporation and connected to the precise multimeter (Keithley 6517B, Cleveland, TX, USA) and computer with data recording for online measurement of the resistance within light-induced cycles. The values of the sensing were calculated according to the following procedure: Resistivity without light stimulation was divided by resistivity upon light stimulation. Temperature during the measurement was checked by external sensor and during the whole investigation was 25 °C ± 0.4 °C.

## 3. Results

### 3.1. SI-ATRP Grafting of GO with PBMA Shell Layer 

**Figure 1:**
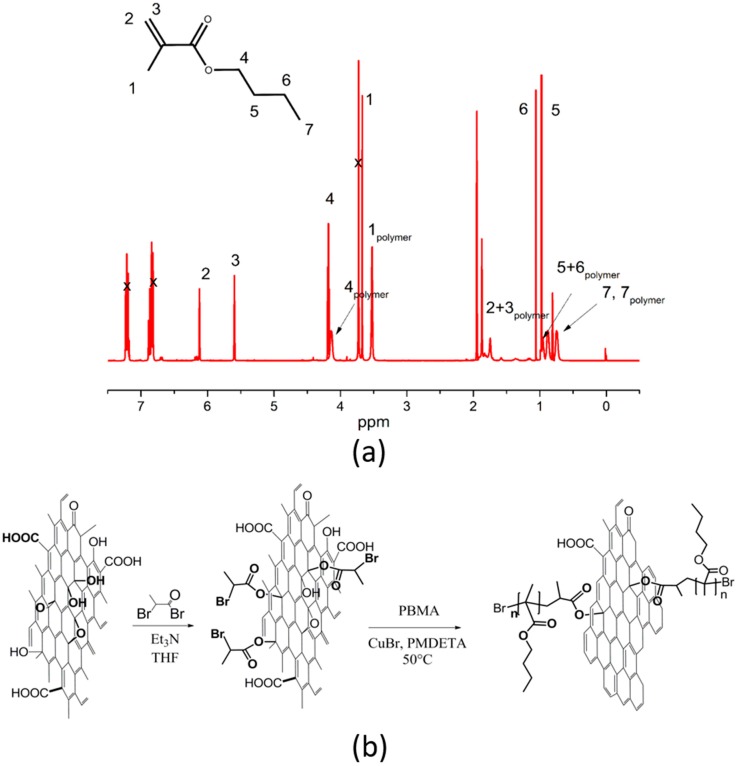
^1^H NMR spectrum for GO-PBMA reaction mixture at the end of polymerization (**a**) and schematic illustration of the initiator grafted to the surface and modification of PBMA via SI-ATRP technique (**b**).

GO particles were modified with PBMA shell layer in the form of polymer brushes using SI-ATRP. Molecular weight was investigated using GPC analysis; namely, Mn of PBMA polymer brushes was 5210 g·mol^−1^ and polydispersity index was PDI 1.21. Monomer conversion was calculated from ^1^H NMR to be 89% and the NMR spectrum for polymer mixture containing both monomer as well as resulted polymer can be seen in the upper part of Figure 1. The schematic illustration based on the Lerf–Klinowski model for GO sheets [44] and the following grafting procedure are visible in the bottom part of Figure 1.

**Figure 2:**
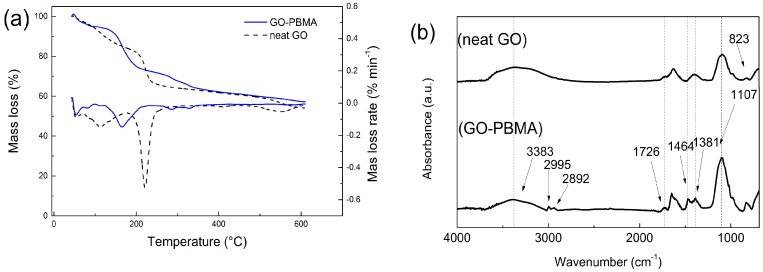
TGA spectra (**a**) and FTIR spectra (**b**).

The successful grafting process was also investigated by TGA (Figure 2a) as well as FTIR in ATR mode method (Figure 2b). As is shown in Figure 2a, the neat GO has two main decomposition regions. First, starting from 75 °C to 180 °C and corresponding to adsorbed water on the GO surface. The second region is from 185 °C to 260 °C and corresponds to oxygen-containing groups. For the GO-PBMA hybrid particles, there is significantly lower adsorbed water due to the substantial polymer coating and region corresponding to oxygen containing groups is shifted to lower temperatures, which is a common phenomenon already published elsewhere [19]. The presence of PBMA coating is visible in the region from 250 °C to 350 °C when the decomposition occurs. The PBMA modification was elucidated by FTIR spectra. In Figure 2b, for neat GO, the absorption band at 3383 cm^−1^ shows the OH groups as a result of successful graphite layers exfoliation and 823 cm^−1^ reflecting the epoxy groups present in the spectra as an absorption band of other oxidation moieties of the GO surface. In the case of GO-PBMA hybrid particles, the presence of polymer is represented by the main absorption band reflecting the presence of methacrylate at 1726 cm^−1^ with butyl vibrations at 1464 cm^−1^ and alkyl chain vibrations at 2995 and 2892 cm^−1^. The ester moieties were found at 1381 and 1107 cm^−1^, showing the proper modification of GO by PBMA brushes.

**Figure 3:**
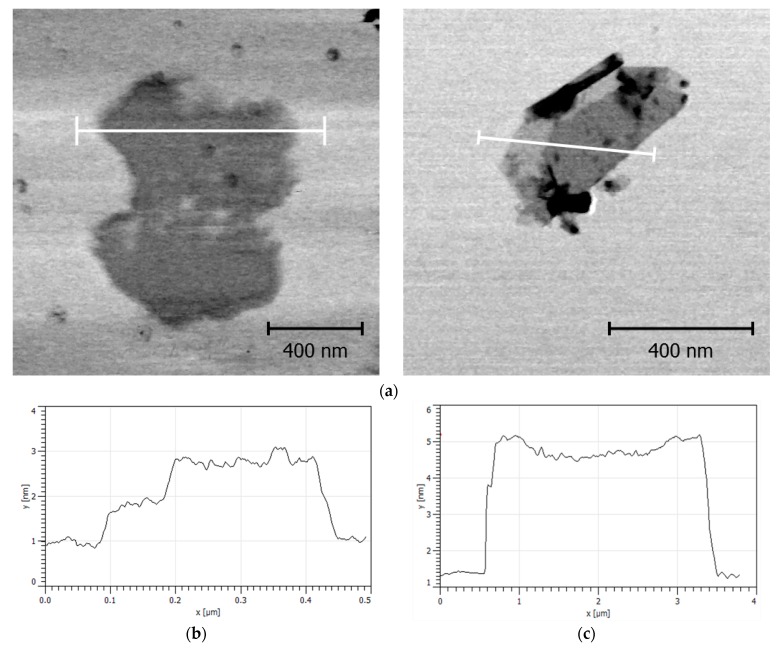
AFM images (**a**) and corresponding thickness profiles for neat GO (**b**) and GO-PBMA (**c**).

In order to confirm the successful exfoliation of the GO particles from the graphite powder and also the successful modification with polymer layer, AFM microscopy was performed. Figure 3 shows that neat GO possess thickness of 1 nm corresponding to a well-exfoliated system with very sharp edges. After the modification with the polymer layer of short polymer chains, the thickness of the sheet increases to 5 nm and edges are significantly less sharp, confirming the successful modification.

**Figure 4:**
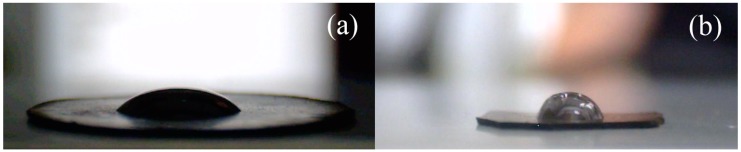
Contact angle measurements of the sessile drop of distilled water; (**a**) neat GO and (**b**) GO-PBMA.

Furthermore, the presence of polymer layer on the GO surface can be confirmed using contact angle investigations. As is shown in Figure 4a, the neat GO shows the contact angle of the water drop to be approximately 43°, while after the PBMA modification, the contact angle increased to 76°, showing the enhanced hydrophobicity, due to the reduced surface of GO as well as to the coating with PBMA. The contact angle is also often used as a measure of compatibility between the particles and matrix. In this case, the increase of the contact angle confirms the better compatibility between the GO-PBMA and PVDF-co-HFP matrix.

### 3.2. Reduction of the GO Particles during SI-ATRP 

**Figure 5:**
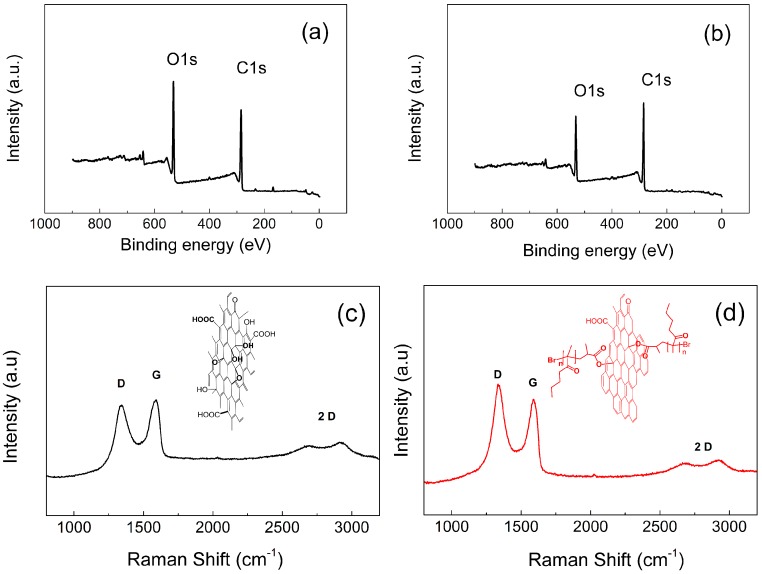
XPS spectra for neat GO (**a**) and GO-PBMA (**b**) and Raman spectra (**c**,**d**) for the same.

Reduction of the GO particles is a very important factor in the case of sensing capability. Therefore, the, XPS and Raman spectroscopy was utilized to investigate the change of sp^2^ and sp^3^ hybridized forms of carbon atoms present in GO. As is shown in Figure 5, there is considerable change between the peak intensities O1s and C1s after SI-ATRP procedure for both neat GO and GO-PBMA particles. All results of XPS investigation are summarized in Table 1. The ratio C/O was significantly changed and clearly shows the reduction of GO as well as the increasing number of sp^2^ carbons corresponding to the regenerated graphene structure. From the Raman spectra (Figure 5), it is clearly shown that the 2D structure of GO is negligibly affected by polymer modification, while the considerable reduction of the GO surface was reached. This reduction was elucidated according to the calculations of the peak intensities those corresponding to sp^2^ and sp^3^ hybridization and are marked I_D_ and I_G,_ respectively. This ratio is for neat GO 0.90 and for GO-PBMA 1.05, showing a usual increase when reduction of the GO surface takes place. Conductivity investigations show that neat GO having 1 × 10^−8^ S·cm^−1^ and GO-PBMA particles has 2.1 × 10^−6^ S·cm^−1^, respectively. Thus, it is concluded that during the SI-ATRP process, reduction of the particles and their simultaneous modification with PBMA polymer chains can be achieved and particles of various conductivities can be prepared.

### 3.3. Preparation of the Non-Woven Fiber Mats

**Figure 6:**
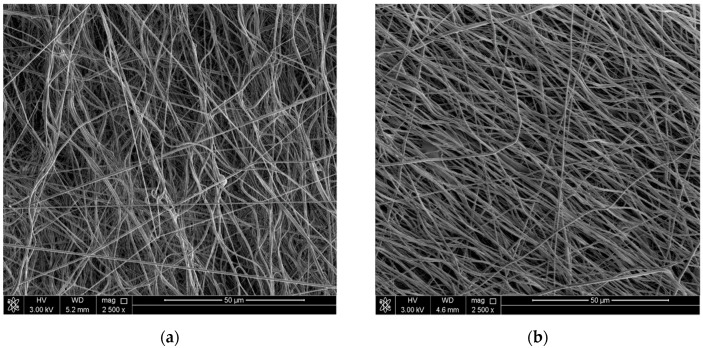
Images from SEM for neat GO (**a**) and GO-PBMA (**b**) based composite systems and their electrospun mats.

As is shown in Figure 6, the electrospinning process was not significantly influenced by GO particles, due to the fact that according to the AFM investigations, the particles are not more than 500 nm wide and 1 nm thick. Therefore, fibers presented in this study have dimensions comparable to those already published [37]. GO with the substantial layer of PBMA on the surface have no impact on fiber processing, because the dimensions of the GO sheets are changed only slightly, but preferential orientation can be attributed to improved conductivity due to the GO-PBMA particles reduction, which considerably enhanced processability of the fiber mats using electrospinning. 

### 3.4. Characterization of the Non-Woven Fiber Mats

**Figure 7:**
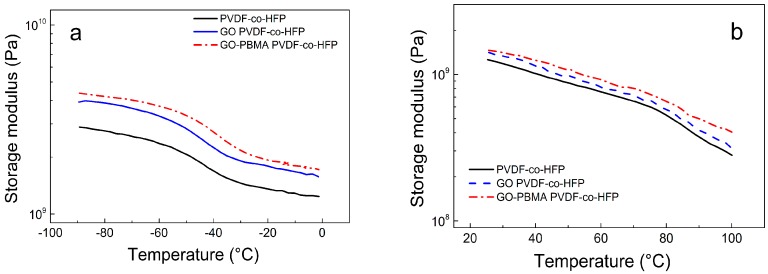

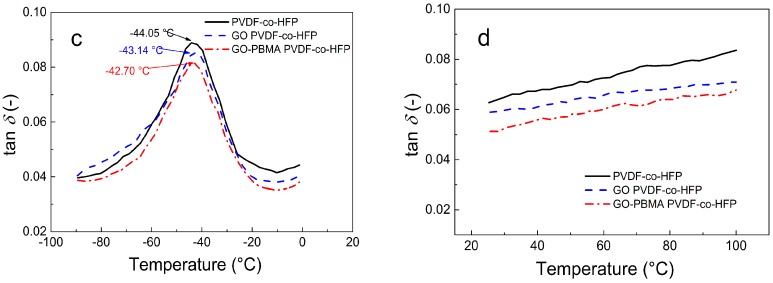
Dynamic mechanical analysis of non-woven fiber mats at temperatures from −100 to 100 °C upon 1 Hz and 0.01% strain deformation, where (**a**,**b**) are storage moduli and (**c**,**d**) are tan δ.

Since, the intended application of the prepared fiber mats is the photo-actuation, which is the dynamic process of reversible actuation upon certain force and frequency, the DMA investigations have been performed. Moreover, the position of the glass transition can also provide additional information about polymer chain mobility as we published previously [5]. Storage modulus represents reversible energy given by material upon dynamic mechanical stimulation, which is an important factor for elucidation of the mechanical performance. As is shown in Figure 7a, the values of storage moduli are very similar in the whole range of investigated temperatures and sustained in a suitable range up to 100 °C (Figure 7b). Influence of the PVDF-*co*-HFP polymer chain mobility can be seen in Figure 7c, where the position of the peak for neat PVDF-*co*-HFP is −44 °C, and with further addition of the GO and GO-PBMA sheets, increases to −43.1 and 42.7 °C, respectively. This indicates that more flexible chains belonged to the neat matrix, however the utilization of systems such as photo-actuators is mostly in the range of 25 °C to 80 °C, when applied as parts of electronics. Therefore, Figure 7d presents this range and it can be seen that tan δ for neat matrix as well as GO and GO-PBMA showed similar values, from 0.06 to 0.08 °C. Such values are very similar to those obtained for other systems with photo-actuation capability. 

### 3.5. Thermal Conductivity Investigation

Thermal conductivity is a very crucial factor for the material actuation upon light stimulation, due to the fact that absorbed light from the source needs to the redistributed within the whole sample and thus provides significantly improved actuation capability [45]. Table 2 shows the values of the thermal conductivity of various non-woven fiber mats. As was already confirmed by Raman spectroscopy and electric conductivity investigations, the electric conductivity of these system increased, which is also in correlation with thermal conductivity, which increased from 0.19 for neat matrix to 0.22 and 0.28 W·mK^−1^ for GO-filled and GO-PBMA filled PVDF-*co*-HFP, respectively. Such increment is very promising from the photo-actuation point of view and will be further presented in Section 3.6 below.

### 3.6. Photo-Actuation and Sensing Capabilities

**Figure 8:**
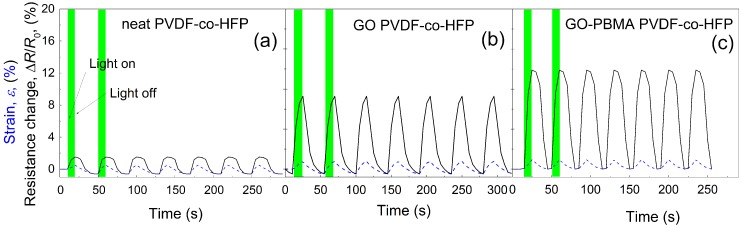
Light-induced actuation strain (dashed blue lines) and resistance change of non-woven electrospun mats (solid black line) at a light intensity of 6 mW for neat PVDF-*co*-HFP (**a**), GO PVDF-*co*-HFP (**b**) and GO-PBMA PVDF-*co*-HFP (**c**).

In order to provide information on how the prepared systems based on PVDF-*co*-HFP fiber mats behave upon light stimulation, the data are presented in Figure 8. Due to the certain flexibility of the polymer matrix as well as non-zero absorption of PVDF-*co*-HFP at 627 nm, the contraction showing the 0.33% strain deformation was achieved. Due to the relatively good electrical properties of PVDF-*co*-HFP, very low resistance change was obtained (less than 2%). After addition of the neat GO sheets to PVDF-co-HFP, the sample exhibited good photo-actuation performance; around 0.83% strain deformation, however the sensing performance was still less than 10%. Moreover, the presence of neat GO prolonged the recovery time significantly, most probably due to the improper GO distribution in the PVDF-*co*-HFP matrix. Furthermore, addition of the GO-PBMA sheets to PVDF-*co*-HFP, which enhanced thermal conductivity, also caused the actuation upon light stimulation to be significantly higher; approximately 1.2% deformation showing values four-fold higher in comparison to neat PVDF-*co*-HFP. Improved electric performance, which comes from simultaneous reduction of GO, also improved the sensing performance, which finally reaches values of sensing around 12.37%.

**Figure 9:**
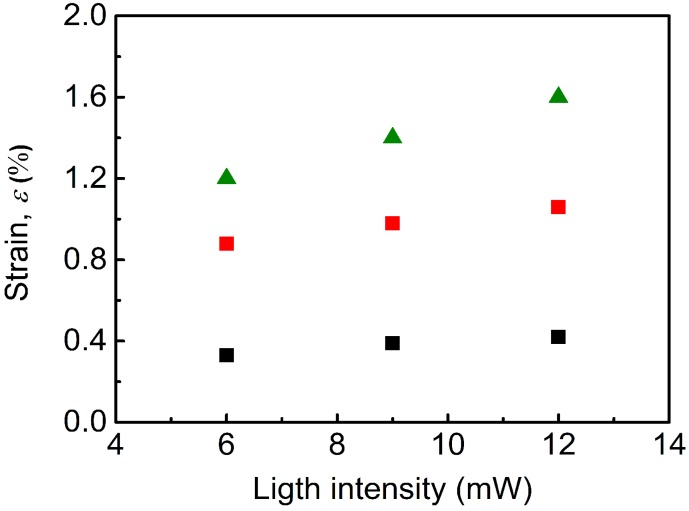
Photo-actuation strain as a function of the light intensity.

Figure 9 presents the effect of the various intensities on the strain deformation. Here, it can be clearly seen that higher intensity showed higher strain deformations. While for neat non-woven fiber mat the increment is very small, from 0.33% to 0.42%, for the GO-PBMA-based fiber mat, the increase is from 1.2% for 6 mW to 1.62% for 12 mW light intensity. This indicates the good capability of the prepared material, since such actuation can be reversibly changed over the time. The calculated strain deformations upon light stimulation are, basically, 10 times higher, in comparison to the already published results by Osicka et al. for same grafts, but in PDMS-based elastomer [11], and 5 times higher than GO-PGMA grafts in PDMS elastomer [39], all at 6 mW light intensity. 

## 4. Conclusions

The presented paper deals with modification of the GO sheets using controllable grafting of PBMA from the surface and simultaneous reduction of the GO in one step reaction. Prepared particles were characterized by various techniques such as FTIR, Raman spectroscopy, XPS, and conductivity investigation. The neat PVDF-*co*-HFP, neat GO, and GO-PBMA based PVDF-*co*-HFP were further used as materials for electrospinning in DMF solution and characterized using SEM.

Dynamic mechanical properties were investigated using DMA and showed that mechanical performance for all investigated systems is in the proper range to be reversibly actuated over the time. Finally, the light stimulation of the presented samples during seven cycles showed that procedure of simultaneous coating and reduction of GO sheets applied in PVDF-*co*-HFP fiber mats exhibits excellent photo-actuation performance as well as sensing capability sufficient for intended applications.

## Figures and Tables

**Table 1:** The chemical composition on the surface of neat GO, GO-I and GO-PBMA. All values are in atomic %.

Sample Name	C1s	O1s	C1s sp^2^	C1s sp^3^	C1s C–O	C1s C=O	C1s O–C=O	C1s/O1s
GO	66.7	33.3	26.7	28.4	32.3	9.0	3.6	2.00
GO-PBMA	70.9	29.1	36.5	25.3	27.5	7.7	3.0	2.43

**Table 2:** Thermal conductivity of various non-woven fiber mats.

Sample Name	Thermal Conductivity (W·mK^−1^)
PVDF-*co*-HFP	0.19
GO PVDF-*co*-HFP	0.22
GO-PBMA PVDF-*co*-HFP	0.28
